# Genetic Polymorphisms Associated with Rheumatoid Arthritis Development and Antirheumatic Therapy Response

**DOI:** 10.3390/ijms21144911

**Published:** 2020-07-11

**Authors:** Dmitry S. Mikhaylenko, Marina V. Nemtsova, Irina V. Bure, Ekaterina B. Kuznetsova, Ekaterina A. Alekseeva, Vadim V. Tarasov, Alexander N. Lukashev, Marina I. Beloukhova, Andrei A. Deviatkin, Andrey A. Zamyatnin

**Affiliations:** 1 Institute of Molecular Medicine, Sechenov First Moscow State Medical University (Sechenov University), 119991 Moscow, Russia; nemtsova_m_v@mail.ru (M.V.N.); bureira@mail.ru (I.V.B.); kuznetsova.k@bk.ru (E.B.K.); ekater.alekseeva@gmail.com (E.A.A.); alexander_lukashev@hotmail.com (A.N.L.); beloukhovamarina@gmail.com (M.I.B.); andreideviatkin@gmail.com (A.A.D.); 2 Laboratory of Epigenetics, Research Centre for Medical Genetics, 115478 Moscow, Russia; 3 Department of Pharmacology and Pharmacy, Sechenov First Moscow State Medical University, 119991 Moscow, Russia; tarasov-v-v@mail.ru; 4 Martsinovsky Institute of Medical Parasitology, Tropical and Vector Borne Diseases, Sechenov First Moscow State Medical University, 119435 Moscow, Russia; 5 Belozersky Institute of Physico-Chemical Biology, Lomonosov Moscow State University, 119992 Moscow, Russia

**Keywords:** rheumatoid arthritis, polymorphism, genetic predisposition, mutation, methotrexate, interleukin, targeted therapy

## Abstract

Rheumatoid arthritis (RA) is the most common inflammatory arthropathy worldwide. Possible manifestations of RA can be represented by a wide variability of symptoms, clinical forms, and course options. This multifactorial disease is triggered by a genetic predisposition and environmental factors. Both clinical and genealogical studies have demonstrated disease case accumulation in families. Revealing the impact of candidate gene missense variants on the disease course elucidates understanding of RA molecular pathogenesis. A multivariate genomewide association study (GWAS) based analysis identified the genes and signalling pathways involved in the pathogenesis of the disease. However, these identified RA candidate gene variants only explain 30% of familial disease cases. The genetic causes for a significant proportion of familial RA have not been determined until now. Therefore, it is important to identify RA risk groups in different populations, as well as the possible prognostic value of some genetic variants for disease development, progression, and treatment. Our review has two purposes. First, to summarise the data on RA candidate genes and the increased disease risk associated with these alleles in various populations. Second, to describe how the genetic variants can be used in the selection of drugs for the treatment of RA.

## 1. Introduction

Rheumatoid arthritis (RA) is a chronic autoinflammatory disease affecting connective tissue, characterised by progressive joint damage and specific systemic disorders. To date, the number of patients with autoimmune arthritis is almost 1% of the world’s population, establishing an urgent problem for healthcare systems worldwide. An evident feature of RA is clinical polymorphism, represented by a wide variability of symptoms, clinical forms, and progression rates. RA is considered to be a multifactorial disease triggered by a genetic predisposition and environmental factors. Both clinical and genealogical studies have shown that the disease can accumulate in families. This relationship has been confirmed by modern molecular genetics. The opportunity to identify RA risk groups in different populations, as well as the possible prognostic value of some genetic variants for disease development, progression, and treatment, including a personalised antirheumatic therapy response, has promoted new studies of germline genetic variants in RA patients [1,2]. This review has two purposes. First, to summarise the data on the RA candidate genes in various populations and the increased disease risk associated with those alleles. Second, to describe how the genetic variants can be used in the selection of drugs such as methotrexate, interleukin-6 signalling pathway inhibitors or TNF (tumor necrosis factor) inhibitors for the treatment of RA. Papers in the PubMed and Scopus databases published in 2010–2020, as well as clinical recommendations and information materials from the American College of Rheumatology and the European League Against Rheumatism, were analysed to understand the current state in this field. A total of 125 sources were selected, of which 96 are mentioned in this review.

## 2. Genetic Predisposition for Rheumatoid Arthritis

The mechanisms for RA development are similar to those of other autoimmune diseases—an immune response to the patient’s self-proteins develops, which drives a cascade of proinflammatory signalling pathways and activates the production of corresponding cytokines and chemokines. In some cases, rheumatoid factor (RF) and anticitrullinated protein antibodies (ACPAs) can be found in patients before any clinical manifestations of RA. Proposed in 2010, the RA classification is based on the presence of ACPAs, found in 90% of patients. These antibodies are considered as more distinctive immunological markers of RA than others, while citrulline compounds seem to play an important role in the disease pathogenesis [3,4].

Unfavourable alleles of various genes have a relatively small influence on the disease risk when they appear individually, but in combination, they genetically predispose an individual to RA development. Currently, more than 100 loci associated with RA have been described [5]. These changes can also be found in genes whose products are not directly involved in immune reactions. The variants associated with RA pathogenesis can be found in a wide range of genes that mediate cell signalling pathways.

### 2.1. Role of HLA Genes in Rheumatoid Arthritis Development

Based on data from the genomewide association study (GWAS), the main genes for RA predisposition belong to the major histocompatibility complex (MHC) locus. This locus, spanning 4 Mbp, occurs in the 6p21.3 region and contains about 250 genes coding the antigen proteins for T-cell recognition. Up to 60% of possible polymorphisms (genetic variants) responsible for RA susceptibility are considered to arise from the MHC locus [6]. For instance, HLA-DRB1 alleles encode unfavourable variants of the shared epitope (SE), which is critical for the correct T-cell antigen presentation process. Eighteen percent of the ACPA-positive and 2.4% of the ACPA-negative RA hereditary component is associated with these alleles. Predisposing HLA-DRB1 SE alleles are found in 64–70% of RA patients and 55% of their healthy relatives, while only being found in 35% of the average population [7].

Sequencing of the HLA-DRB1 alleles encoding the polymorphic β-chain of the DR molecule shows a prevalence of *04:01 and *04:04 alleles in RA patients in Europe and the *04:05 allele in East Asia. Alleles *04:02 and *04:03 are shown to be protective. Based on these data, the hypothesis of the importance of the conserved epitope structure, which forms the third β-chain hypervariable region (positions p.70-74) for RA development, was postulated: whether it contains the unfavourable variants, i.e., QKRAA, QQRAA, KKRAA, QRRAA, RRRAA, or, on the contrary, the protective structure of DERAA. Moreover, amino acid substitutions at positions 11 and 13 of DR4 are also associated with RA (alleles *04:01/*04:04/*04:05 compared to DR1 *01:01) [1,8,9]. Association of the ACPAs and the hypervariable epitope alleles indicates the role of these variants in the T-cell presentation of citrullinated proteins and the further development of autoreactivity. In addition, some of the specific positions of amino acid residues suggest their participation in HLA-DR intracellular transport. Citrulline residues are located inside the DRB1*04:01/04 positively charged P4 pocket of the peptides but not in the negatively charged P4 pocket (encoded by the protective allele *04:02), as shown by model experiments and crystal structure analysis. Namely, the P4 amino acid residues at positions 13 and 71 directly interact with the citrulline, so their alterations are believed to be the most relevant for RA development [10,11].

As noted above, polymorphisms leading to substitutions of amino acid residues at positions 11, 13, 71, and 74 of HLA-DRB1, as well as at position 9 in HLA-B and HLA-DPB1, have the greatest association with RA development. These residues are faced towards the protein pocket to bind the antigen in HLA molecules. These relations are more evident in African Americans and Asians compared to Caucasians. In contrast, HLA-DR3 associations, mainly the protective role of the HLA-DRB1*13 haplotype (five amino acid residues of the “wildtype” DERAA at positions 70–74), are shown in European populations. Individuals of such a haplotype have a decreased number of autoreactive T-cells, presumably due to their negative thymus selection [6]. Associations with HLA-DRB1 alleles at position 57 rather than 71–74 are shown in African populations [12]. Thus, the main critical site related to RA development in different populations of both Caucasians and Asians is the 13th amino acid residue. This position substitution increases its pathogenic effect as follows: Ser < Gly ≤ Tyr < Arg < Phe < His [8]

The observed data explain the hypervariable epitope and its polymorphism roles in the development of RA with ACPA formation. However, HLA-RA associations are shown in the absence of ACPAs as well. The DRB1*03:01 allele associated with ACRA-negative RA was found in Europeans, but not Japanese, in whom HLA-DR8 and DR14 homozygotes are associated with the disease. In addition, DRB1*03 (serine at position 11) and HLA-B*08 (aspartic acid at position 9) alleles are associated with ACPA-negative RA regardless of population. Hence, RA is not only clinically but also genetically a heterogeneous disease [13].

In general, RA-associated amino acid positions are located in the middle of the HLA molecules epitope-binding pockets; thus, the substitutions can directly affect antigenic specificity and result in protein binding to the citrulline residues. In discrepancy with the Hardy–Weinberg ratio, heterozygotes are more common in patients with RA (they have a greater predisposition to ACPA-positive RA). This could be due to a wider range of exposed autoantigens compared to homozygotes [14]. Citrullinated proteins are seen as foreign antigens. This complies with the confirmed hypotheses of neoantigens (i.e., peptides with citrulline residues), as well as with the assumption that the calreticulin signalling pathway and the low-affinity HLA-DR molecules carrying SE epitopes for class II-associated invariant chain peptides (CLIPs) are involved in RA pathogenesis [10,15].

In addition to the HLA-DRB1 alleles, other loci have also been associated with RA development, particularly the *04 allele of the DQB1 locus, as revealed by meta-analysis [16]. It is not only the polymorphisms predisposed to RA that are of interest to professionals in rheumatology, but also those associated with disease prognosis and the severity of the disease. Thus, the HLA-DRB1*08, HLA-DRB1*11, and HLA-DPB1*02:01 alleles are associated with oligoarticular RA—no more than 4 points, according to the International League of Associations for Rheumatology (ILAR) classification. Polyarticular RA (5 points or more) is associated with the HLA-DRB1*11:01 and HLA-DRB1*11:04 alleles [10]. At the same time, a meta-analysis of 2775 RA cases showed the association between HLA-DRB1*04:01 polymorphism and the degree of joint damage [17]. The combination of HLA gene alleles predisposed to RA predetermines the genetic component for the development of this disease. At the same time, many other genes encoding enzymes, receptors, and auxiliary proteins may affect the severity of the clinical manifestations and the development of RA [18].

### 2.2. Polymorphisms of Non-HLA Genes Associated with Rheumatoid Arthritis

Genes for the predisposition to RA can be conditionally divided into groups of cytokines and their receptors, chemokines and their receptors, key components of intracellular signalling pathways, and auxiliary (costimulating) factors.

#### 2.2.1. Cytokines and Cytokine Receptors

A significant number of RA-associated genetic variants have been identified in the interleukins (ILs) and their receptor genes. Interleukins are cytokines that stimulate hematopoietic cell development and T- and B-lymphocyte differentiation. There are seven interleukin families characterised by significant variability in both ligand forms and receptors. The key mediator of inflammation, IL1, is represented by two isoforms and is combined with another nine interleukins in one family. Its expression is affected by the –511A/G (rs16944) polymorphism in the promoter (associated with RA in Caucasians) and the +3953C/T polymorphism in exon 5 (RA-associated in some Asian populations). According to meta-analyses, RA-associated alleles in Caucasians include –174G/C and –572G/C of the *IL6* gene, rs1800896 in the *IL10* gene, rs13151961 and rs6822844 located near the *IL2* and *IL21* genes, rs7530511 and rs11209026 in the *IL23R* gene, and some others. A significant number of RA-associated polymorphic loci included in the meta-analyses are unique to Asian populations. The polymorphism found at rs1946518 of the *IL18* gene is found in Egyptian populations, and the polymorphism found at rs549908 is found in the Taiwanese population. Thus the candidate genes for assessing genetic RA predisposition need to be thoroughly selected [19,20,21,22].

Another candidate gene is *IL2RA*, which encodes the interleukin 2 (IL2) receptor α-subunit. The rs12722495 polymorphism of this gene correlates with its mRNA and protein expression in stimulated monocytes, naive T-cells, and memory T-cells [6]. Additionally, according to a meta-analysis of 37 studies, a minor allele A of the rs1343151 polymorphism in the IL23R gene, which encodes the receptor for interleukin 23, is associated with a risk of developing RA [23].

#### 2.2.2. Chemokines and Chemokine Receptors

Changes in the *CCR6* gene can affect the development of RA. This gene encodes the chemokine receptor, which is the surface marker of Th17 cells. Dinucleotide polymorphism rs3093024 in the 5′ regulatory sequence of CCR6 binds nuclear proteins. This polymorphism is associated with expression levels of the chemokine receptor and increased plasma concentration of IL17 cytokine in RA patients. The role of the CCR6 receptor in RA development appears to be more significant than previously known due to its expression on different subtypes of T-cells involved in the regulation of inflammation. According to a meta-analysis, rs3093024 and several other SNPs (single nucleotide polymorphism) in *CCR6* are associated with RA in European and Asian populations [6,24]. At the same time, a 32-bp deletion in another chemokine receptor gene, *CCR5,* has a protective effect, according to a case–control study in Brazil with samples of 740 RA patients and 676 controls [25].

The *CCL2* gene encodes the chemoattractant protein, which recruits monocytes and T-cells during inflammation. A meta-analysis of the functionally significant 2518A/G polymorphism of this gene promoter on chromosome 17 was performed. This polymorphism was studied for associations with various autoimmune diseases (systemic lupus erythematosus, RA, systemic sclerosis, nephropathy with Ig-A, Crohn’s disease) in different populations. The unfavourable allele A showed the most pronounced association with RA in Asian populations, as well as with Crohn’s disease in Europeans [26].

#### 2.2.3. Components of Intracellular Pathways That Regulate Proliferation

A number of RA candidate genes are involved in the NF-κB signalling pathway. One of the *CD40* alleles, which is also an RA candidate gene, is associated with increased expression of CD40 molecules on the surface of B-cells and intensified NF-κB signalling. Another RA candidate gene is *TNFAIP3*, which encodes a component of the NF-κB signalling pathway—the A20 ubiquitin-modifying enzyme. These independent gene polymorphisms associated with RA were identified by meta-analyses. One of them—the TT > A substitution located a 42-kb distance from the promoter—reduces the binding avidity of NF-κB signalling pathway transcription factors, leading to decreased TNFAIP3 and A20 protein expression. Another possible SNP is the replacement of phenylalanine with cysteine at the 127 position, which disturbs the A20 function. Interestingly, A20 is often inactivated due to somatic mutations in B-cell lymphoma. The *TNFAIP3* gene is located in the 6q23 region. Besides the *TNFAIP3* gene, this region contains plenty of autoimmune disease candidate genes: *IL20RA*, *IFNGR*, *OLIG3* and others [27,28].

Involved in the JAK/STAT pathway, the *STAT4* gene was identified as a candidate gene for RA and other autoimmune diseases as a result of GWAS. *STAT4* encodes the signal transducer and activator of transcription protein 4 (STAT4). The rs7574865 polymorphism of the *STAT4* gene is associated with RA in different ethnic populations. The STAT family proteins act as JAK-activated transcription factors and are important in many cytokines signalling pathways, primarily the interferon pathways. Detected unfavourable rs7574865 alleles were associated with STAT4 overexpression and increased production of interferon-α [29]. STAT4 transcription factors are also involved in the regulation of proinflammatory helper T-cell (Th1, Th2, Th17) differentiation, of which Th1 and Th17 are directly involved in autoimmune reactions [6,19,29].

#### 2.2.4. Genes That Encode Costimulatory Molecules

Some of the most significant RA candidate genes are enzymes or costimulating auxiliary proteins that are important for the metabolism of immunocompetent cells, although not directly involved in the formation of the immune response. For instance, a missense mutation in exon 14 of the *PTPN22* gene leads to the arginine/tryptophan replacement at the 620 position of the polypeptide chain (p. R620W, rs2476601) [30]. This R620W substitution is localised in the motif responsible for the interaction of cofactor proteins and could mediate an increase in autoreactive B-cell numbers during the experiment. This replacement is not only associated with RA but other autoimmune diseases as well, namely, systemic lupus erythematosus, type 1 diabetes, and Graves’ disease (European populations only; the site is usually not polymorphic in Asians). The *PTPN22* gene encodes lymphocyte tyrosine phosphatase, and the mentioned substitution acts as a gain of function mutation. A detailed study of the normal and mutant alleles’ functions in model experiments revealed unexpected difficulties—the homologous 619W mutation in mice acts as a loss of function mutation that is identical to the full knockout of the orthologous gene [6,19,30]. According to a recent meta-analysis of 53 original case–control studies, the polymorphism rs2476601 is associated with RA development in homozygous and heterozygous models in Caucasians and Africans [31]. In addition, the C allele of rs2488457 is associated with RA progress in Caucasians but not in Asians [32]. PTPN22 interacts with the product of *PADI4*, another RA candidate gene encoding peptidyl arginine deaminase. This enzyme converts arginine amino acid residues into citrulline, and a disturbance of this process can contribute to anticitrulline antibody formation. Increased PADI4 activity leading to abnormal citrullination of fibrin strands is noted in inflammatory infiltrate. In addition, it is known that PADI enzyme expression is increased and citrullinated peptides are found in the bronchoalveolar lavage cells of smokers compared to nonsmokers. Smoking is considered an RA-provoking environmental factor that contributes to the multifactorial basis for disease development [33]. The *PADI4* gene located in the 1p36 region was the first RA candidate gene identified in the Asian population. Unfavourable substitutions of the *PADI4* codons—G55S, V82A and G112A—do not significantly affect the activity of the enzyme itself but reduces the gene’s mRNA stability. A meta-analysis of the SNP gene in 20,000 RA patients and 25,000 controls determined the associations of the disease with the –94G/A polymorphism in Asian populations, the –92C/G polymorphism in African populations, and the -–90C/T polymorphism in Latin populations. Interestingly, the PTPN22 and PADI4 interaction most likely has a functional significance since PTPN22 deficiency leads to an increased production of citrulline compounds and the formation of extracellular neutrophilic traps [6,27,34].

The autoimmune regulator transcription factor (AIRE), with its 11 polymorphic variants studied in autoimmune diseases, should also be noted. The association of rs2075876 and rs760426 of *AIRE* gene polymorphisms with RA was shown by the meta-analysis, which included a total of 6696 RA patients and 8164 controls. The polymorphisms were localised in the gene promoter; the presence of unfavourable alleles reduced gene expression. A lack of AIRE protein led to the failure of naive T-cell specific selection in the thymus, resulting in autoimmune T-cell survival [35]. Thus, specific polymorphisms associated with the disease as predisposition factors in various populations and heterogeneous clinical groups, according to a number of meta-analyses, could be distinguished among the large number of genetic variants related to RA. The main RA-predisposing polymorphisms are presented in Table 1.

Some of the candidate genes and polymorphic variants are not directly related to cytokines and intracellular signalling pathways that enhance inflammatory processes. However, polymorphisms in such genes may change the activity of the protein product, which leads to the accumulation of certain metabolites and the subsequent overproduction of proinflammatory cytokines. For example, a meta-analysis shows the association of intron SNP in the *SLC8A3* gene encoding a Na^+^/Ca^+^ transmembrane transfer protein with ACPA-positive RA. However, there is evidence that this carrier protein hyperactivity may be accompanied by an increased level of tumour necrosis factor alpha (TNFα) [47]. The *MTHFR* gene encodes methylenetetrahydrofolate reductase, promoting the homocysteine to methionine conversion that is important for folate metabolism and the synthesis of nucleic acids. According to the meta-analysis, the T-allele of the C677T polymorphism (rs1801133) is associated with RA in Caucasians. This allele encodes an enzyme with reduced activity that leads to hyperhomocysteinemia in homozygotes, which results in an increase in proinflammatory cytokine concentration [40]. Moreover, according to a meta-analysis of 16 original studies published in 2020, C677T is associated with RA in the dominant and recessive models in Caucasians and Africans, and in the recessive model in Asians [41]. In addition, a meta-analysis of eight papers demonstrated the association of TIM3 with RA development. This gene encodes the auxiliary factor expressed by dendritic cells, macrophages, type 1 T-helper, and type 2 T-helper cells. TIM3 modulates the differentiation of type 1 T-helper and type 17 T-helper cells, which suppress autoimmune processes. The polymorphism rs1036199 in TIM3 is associated with RA development [48].

## 3. Predisposition to RA Depends on the Method of Analysis of Genes and Population Characteristics

Candidate gene missense variant impact studies have led to a greater understanding of the molecular pathogenesis of RA. A multivariate GWAS-based analysis identified the genes and signalling pathways involved in the pathogenesis of the disease. However, these identified RA candidate gene variants only explain 30% of the disease cases with family accumulation, while the actual family prevalence among all RA patients reaches 65% [1,14,18]. The genetic causes of a significant proportion of familial RA have not been identified until now. Perhaps high-performance sequencing technology—high-throughput sequencing (HTS)—will help to solve this problem in the near future. The list of unfavourable alleles has already been supplemented with missense variants of the *IL2RA* and *IL2RB* genes by the first HTS experiments. By the in-silico constructing of an interactome between the already known RA candidate genes, about 160 other presumptive candidates were identified. Subsequently, a number of them were considered RA-associated according to the GWAS results: the *NOTCH4* gene, the *TNXB* gene located near the MHC class III genes, the *BTNL2* gene (especially rs3817963; also associated with systemic sarcoidosis), as well as the insufficiently characterised sequence C6orf10, expressed in autoimmune and some other diseases [49].

The population genetics characteristic, as well as the results of previous meta-analyses, need to be taken into account when using GWAS and HTS technologies. A list of the main predisposing RA genes identified in Latin American populations is an example of such research. The list was mostly formed based on the results of studies done between 2003 and 2013, and it is consistent with similar lists of European populations. This could be explained by a significant proportion of Spanish origin RA patients amongst all of the examined patients. At the same time, the RA candidate genes attributable to Latin American populations have only been described in recent years, such as *ENOX1* on chromosome 13 and *NNA25* on chromosome 12 [7].

SNPs occur in no more than 1% of the coding part of the genome; most of them are localised in noncoding DNA. Single RA-associated nucleotide substitutions have been detected near the noncoding regions of 40–50 genes. At least some of them are suggested to increase RA risk by means of the activation of tissue-specific superenhancers. Thus, polymorphic variants associated with RA are mainly localised in the T-cell and natural killer enhancers compared to the other cell types, according to the FANTOM5 consortium (contains information for 71 cell type tissue-specific enhancers). In addition, SNPs associated with RA and other autoimmune diseases are more likely to occur in superenhancers than in “regular” enhancer regions of CD4^+^ T-cells [50]. These superenhancers are associated with 27 of the 100 major RA candidate genes. Moreover, 12 loci have shown an association with RA according to GWAS and contained long noncoding RNA genes that regulate the expression of other candidate genes. RA-associated SNPs of noncoding regions are often found in candidate gene enhancer regions. Therefore, a number of functionally significant polymorphisms could be identified by reverse genetics methods. Some authors have searched for SNPs in regulatory regions of genes using data on its expression changes. The RA association of rs2013109 in the *RNASE2* gene was shown this way [51]. Currently, the search for RA candidate genes is performed by bioinformatic analysis of published expression profiles obtained with high-density microchip technology (for instance, Affymetrix) [52,53,54,55]. Whole-genome or whole-exome sequencing, utilising high-performance HTS platforms, could also help to identify rare and population-specific pathogenic genetic variants. In particular, rare RA-associated germline variants were identified in the *NCR3LG1*, *RAP1GAP*, *CHCHD5*, *HIPK2*, and *DIAPH2* genes by whole-exome sequencing with Illumina HiSeq in a Han (Chinese ethnic group) patient cohort [56].

The data presented in this section indicate the significant experimental and bioinformatic work performed by researchers in countries all over the world to elucidate the genetic causes of RA development. However, whether the genetic determinants can help in personalising and increasing the effectiveness of RA treatment remains an important question for clinical rheumatology.

## 4. Genetic Factors are Prognostic Markers Associated with RA Clinical Manifestations

There are currently many genes and loci associated with a predisposition to RA. The identification of genetic markers associated with the clinical prognosis of RA is more difficult than identifying predisposition markers. This is because the severity of the disease depends not only on genetic factors but also on epigenetic changes, as well as the action of provoking environmental factors.

Some genetic factors predisposing to RA can be associated with the intensity of the course of the disease. As such, the genetic variants HLA-DRB1 alleles were identified as associated with radiologically determined joint damage, which is one of the main manifestations of the severity of the disease. Sixteen HLA-DRB1 haplotypes are associated with an increased risk of RA, erosive joint damage, and the patient’s lifespan [57]. Valine at position 11 of HLA-DRB1 is a predictor of joint erosion and an unfavourable outcome, whereas serine at the same position is associated with a less severe clinical course of the disease [58]. Besides such modifications, the rs112112734 polymorphism of the HLA-DRB1 gene is also connected with radiologically determined joint damage [17]. In addition, this study showed that rs112112734 is also associated with the presence of rheumatoid factor and ACPAs, which are serological markers of RA.

The IL6 signalling pathway is an important participant in the proinflammatory network of cytokines, which contributes to the destruction of articular tissues. A study of the IL6R gene polymorphism and the extent of joint erosion in RA revealed an association between SNP rs4845618, located in the first intron of the *IL6R* gene, and joint damage. It was shown that rs4845618 is significant for the expression of IL6R in the study of whole blood samples. Using the latest data from the Roadmap Epigenomics Project epigenetic consortia, it has been demonstrated that the SNP rs4845618 localisation region is associated with the regulation of gene activity in more than 50 different types of human cells, including T-cells. This suggests an association between joint damage and the regulatory sequence of the *IL6R* gene [59].

The serum level of another interleukin, IL37, which is one of the key modulators of RA, correlates with disease activity. An analysis of the *IL37* gene rs3811047 polymorphism in patients with RA in the Egyptian Arab population showed that patients with the GG genotype had a higher severity score of lesions on the DAS28 clinical scale than patients with genotypes AA or AG [60]. In another study, the polymorphism rs911263 located inside the *RAD51B* gene was presented as a genetic factor associated with joint erosion and the severity of RA. Allele C rs911263 is associated with a lower incidence of joint erosion in patients with RA [61]. This polymorphism demonstrated a strong protective effect on RA [61]. RAD51B is a member of the RAD51 family of proteins that are required for DNA repair through recombination. It is currently not known how this polymorphism is functionally related to the severity of the clinical manifestations of RA.

Associated with the development of RA in the Korean population and Caucasians, the *UBASH3A* gene [62] is also associated with the intensity of the course of the disease. The C allele of the rs1893592 polymorphism of the *UBASH3A* gene was shown to be protective with respect to RA activity (scores according to DAS28, C-reactive protein level and bone erosion). At the same time, this may be caused by population specificity [63]. The *UBASH3A* gene encodes a protein involved in the degradation of receptor tyrosine kinases, with proapoptotic properties in T-cells, which may explain its involvement in the pathogenesis of RA. It is noteworthy that AA homozygotes at position –308 of the *TNFA* gene also have a pronounced association with both the risk of developing RA and the severity of the clinical picture of this disease [64].

The above examples show that polymorphic variants of RA candidate genes can not only increase the risk of developing a disease but also increase the likelihood of a more severe course of the disease, ceteris paribus. Although their use in clinical practice as markers is impractical due to low penetrance and, in some cases, population specificity, polymorphisms associated with RA activity indicate the molecular pathways underlying the intensive development of RA and the possibilities of targeted therapy of this disease.

## 5. Genetic Predictors of Response to Rheumatoid Arthritis Drug Therapy

### 5.1. Therapy of RA with the Use of Antirheumatic Drugs

RA treatment aims to achieve clinical remission and minimal disease activity. The classes of drugs used in RA therapy are known under the common name disease-modifying antirheumatic drugs (DMARDs). Synthetic DMARDs are divided into conventional synthetic DMARDs (csDMARD, for example, methotrexate, leflunomide, sulfasalazine, hydroxychloroquine, and others) and targeted synthetic DMARDs (tsDMARD, for example, baricitinib, tofacitinib, upadacitinib). Biological DMARDs (bDMARDs) comprise the second group of drugs. bDMARDs may be subdivided into distinct classes depending on their targets: TNF inhibitors (adalimumab, certolizumab, etanercept, golimumab, infliximab), IL6 inhibitors (sarilumab, tocilizumab), inhibitor of B-cells harboring CD20 (rituximab), and costimulating factors (abatacept). According to the recommendations of The European League Against Rheumatism (EULAR), updated in 2019, some basic rules should be followed in the treatment of RA. DMARD treatment should be started immediately after a diagnosis of RA. The first line of therapy uses methotrexate, which is the most widely used csDMARD. Methotrexate can be effective both in mono mode and in combination with other csDMARDs, tsDMARDs, and bDMARDs. If the patient has methotrexate intolerance, then leflunomide or sulfasalazine may be used instead. Glucocorticoids are prescribed only to a small number of patients for a short period in the first line of therapy. If during the first three months of treatment with methotrexate, there is no improvement, and clinical remission is not achieved after six months, then the second line of RA therapy should be used. In the absence of unfavourable prognosis factors (high levels of ACPAs, rheumatoid factor, high activity of the disease according to clinical scales and early damage to joints, ineffective use of two csDMARDs), another csDMARD is added to methotrexate. Another possible option is to replace methotrexate with another csDMARD. In the presence of unfavourable prognostic factors, bDMARDs or JAK inhibitors may be used instead of methotrexate. If after some time (for example, three months), it is not possible to achieve an improvement in the condition and clinical remission, then another bDMARD is prescribed as a third-line drug. If remission is achieved as a result of using any of the described lines of therapy, the dose of DMARD is reduced and the optimal period of visits to the rheumatologist is established to control the activity of the disease [65]. Similar recommendations for the treatment of RA were previously published by the American College of Rheumatology (ACR) [66]. It should be noted that maybe in the future, the characteristics of the patient’s genome would determine the sensitivity and resistance to various DMARDs.

### 5.2. Genetic Variants Affecting csDMARD Effectiveness

Methotrexate-sensitivity pharmacogenetic studies are quite numerous as it is the first-line therapy drug of choice. The main genetic variants associated with methotrexate treatment response determined by separate original works, multicenter studies, and meta-analyses are summarised in Table 2; similar loci associated with the effectiveness of targeted DMARD therapy are also shown. A better methotrexate response is noted in the presence of 3435C > T polymorphism’s T-allele in *ABCB1* gene exon 26. In contrast, del28-bp TSER*3/*3 homozygotes of the thymidyl synthase gene (*TYMS*) require a higher dose of the drug. Finally, a C > G substitution at position 347 of the *ATIC* gene is associated with methotrexate toxicity. As a less often used first-line drug for RA treatment, leflunomide has a lower response rate among 19AA homozygotes of the *DHODH* gene, encoding an enzyme of the de novo pyrimidine synthesis chain [67]. According to recent meta-analyses and earlier original works, sensitivity to methotrexate is associated with 34C/T substitutions in the *AMPD1* gene, 675T/C in the *ATIC* gene, and 80G/A in the *SLC19A1* gene in Caucasians, while 3435C/T in the *ABCB1* gene in this population and 28-bp 2R/3R in the *TYMS* gene in representatives of other races are associated with resistance. Polymorphisms in the *MTHFR* (C677T and A1298C) and *TYMS* (1494 del6 and 28-bp 2R/3R) genes were associated with the severity of side effects in various populations [11,68,69,70]. The *FPGS* gene encoding the transfer protein has rs10987742 and rs10106 SNPs associated with methotrexate sensitivity in RA [71]. Among the genes encoding other transfer proteins, methotrexate resistance in RA was associated with the *SLC22A11* gene (rs11231809, T-allele), the *ABCC1* gene (rs246240, G-allele; rs3784864, G-allele), and its CGG-(rs35592, rs2074087 and rs3784864) and CGG-haplotypes (rs35592, rs246240 and rs3784864), as well as with substitutions in the *SLC19A1*, *SLC46A1*, and *SLCO1B1* genes [72,73]. However, the last two mentioned studies were performed in a Portuguese population only and may not reflect associations in other Caucasoid groups. It is preferable to focus on meta-analyses that include dozens of original works with Caucasians as an object of study. Thus, the association of the methotrexate toxicity and 80G/A polymorphism in the *RFC1* gene, encoding a transfer protein involved in the folate cycle, was shown [11,74].

Patients could also be prescribed transitional therapy with glucocorticoids, anti-inflammatory drugs that suppress the immune system (prednisone, dexamethasone, methylprednisolone), before the maximum effect of DMARDs is reached. Subsequently, corticoid dose is gradually reduced, and the therapy is transferred completely to DMARDs. The rs37972 polymorphism minor allele of the glucocorticoid-induced transcript 1 gene (*GLCCI1*) was shown to be associated with reduced glucocorticoid sensitivity in men [82]. Polymorphisms associated with these anti-inflammatory drug sensitivity panels include the GLCCI1-C allele, glucocorticoid receptor gene (*GR*) BclI-G (rs41423247) and N363S-G (rs6195) alleles, and the G2677A/T replacement in the multidrug resistance factor (MDR1) *ABCB1* gene [82,87].

### 5.3. Polymorphic Genetic Variants Associated with the Biological and Targeted DMARD Effectiveness

About 45% of patients with RA develop resistance to methotrexate by the end of the second year of treatment. In such cases, the therapy should be switched to targeted drugs. Such second line DMARDs may be inhibitors of the TNFα-signalling pathway, which reduce inflammation and include infliximab, adalimumab, golimumab, etanercept, and certolizumab. These drugs interfere with the binding of TNFα with its receptor. Second-line therapy is quite expensive, while drug resistance occurs in 30–40% of cases. Rituximab, affecting B-cells, or recently developed targeted drugs such as tocilizumab or sarilumab (blocking the IL6-signalling pathway) could be used as a treatment of choice [49,88,89].

Some of the polymorphisms associated with RA affect gene expression or the function of its protein product; hence, the search for targeted drugs based on genetic data is possible. To date, there are specific FDA-approved (Food and Drug Administration) drugs for autoimmune disease treatment targeted against 18 known proteins encoded by RA-associated genes. Among them are abatacept and tocilizumab, which interfere with the *CTLA4* and *IL6R* gene products, respectively. Abatacept, a recombinant protein consisting of the CTLA-4 extracellular domain and the human IgG1 Fc-domain, interacts with the CTLA-4 molecule (CD28) and thereby inhibits T-cell activities. Tocilizumab is a monoclonal antibody blocking the IL6 receptor (IL6R) in soluble form as well as in membrane-bound form.

Since the IL6 signalling pathway is one of the main proinflammatory cascades in RA, its inhibition leads to a pronounced therapeutic effect. However, sensitivity to tocilizumab may vary; for instance, the replacement of asparagine with alanine in the 358 codon of the *IL6R* gene increases the relative concentration of the soluble receptor form by 35%. According to an original RA study in Spain, patients with the AA rs12083537 and CC rs11265618 genotypes respond better to targeted tocilizumab therapy [80]. Tocilizumab efficacy is associated with the *IL6R* gene polymorphisms rs12083537, rs2228145, and rs4329505, as shown by various sources [11]. Tofacitinib, which has been approved by the FDA and EMA (European Medical Agency) to treat RA, could also be considered as a drug of choice. It regulates the inflammatory process in RA by blocking JAK1-3 [13,14]. Other possible options are anakinra, an IL1R inhibitor, or secukinumab, directed against the cytokine IL17. The association of HLA-DRB1*SE (unfavourable alleles in the RA-critical epitope) and HLA-DRB1*pos11 V/L with secukinumab therapy effectiveness was shown during a pharmacogenetic study [90].

Expanding the list of treatment options for RA is important for personalised therapy assignment. Thus, the use of anti-TNF antibodies led to a revolution in RA treatment. However, up to 40% of patients remain resistant to this therapy, as estimated by various authors, although it has been shown to date that some of them may respond to anti-IL6R therapy. This is where the analysis of gene networks in RA can contribute. In particular, rituximab, initially developed as an antitumour agent, has specificity for CD20 molecules and suppresses CD20^+^ B-cell function. As CD20 is encoded by one of the candidate genes associated with RA, therapy with rituximab results in the reduction of disease symptom intensity. Rituximab’s effectiveness is associated with polymorphisms *FCGR3A* rs396991, *IL6* rs1800795, and *BAFF* rs9514828. Moreover, rs4810485 of the *CD40* gene’s allelic variants is associated with increased gene expression. Such CD40 overexpression results in an increased concentration of TNF superfamily receptor type 5 on the blood mononuclear cells’ surface, which makes them targets for TNF inhibitors in this particular cohort of patients [11,14]. Polymorphic variants of the genes encoding TNF and its receptor (*TNFA*), namely, –875T, –308G/A (rs1800629), 238G/A (rs361525), and TNFR1A 36A, are also associated with TNF inhibitor (etanercept, infliximab, and adalimumab) sensitivity [11,91]. Increased anti-TNF therapy sensitivity has also been shown in women with HLA-E*01:03:01/01:03:01 alleles (Polish population only) [92]. *MED15* and *MAFB* genes polymorphisms are associated with etanercept and infliximab sensitivity according to the results of GWAS in Spain [93]. As was shown in a meta-analysis, tyrosine phosphatase receptor type C (*CD45*) and Fc-γ receptor type 2A gene polymorphisms are also associated with anti-TNF drug sensitivity. Thus, the HH + HR genotype of the *FCGR2A* gene is associated with adalimumab efficacy in patients with RA, while the polymorphism rs10919563 allele A of the *PTPRC* gene is related to the sensitivity of three anti-TNF drugs [84,94]. Polymorphisms of the Toll-like receptor genes, which are initial units in proinflammatory intracellular signalling pathways, such as *TLR5* (rs5744174) and *TLR1* (rs4833095), are shown to be associated with the effectiveness of anti-TNF therapy as well [95]. According to the other meta-analyses, more than 20 SNPs associated with the targeted anti-TNF therapy response in patients with RA could be identified in the genes involved in T-cell functioning, NFκB and TNFα signalling pathways, as well as in *CTCN5*, *TEC*, *PTPRC*, *FCGR2A*, *NFKBIB*, *FCGR2A*, *IRAK325*, and other genes [85]. A recently published review summarised data on various polymorphisms of 12 genes, best studied as predictive factors for the response to RA drug therapy: methotrexate (HLA-G, MTHFR, ABCB1, TNFA, TYMS), leflunomide (CYP1A2, CYP2C19), etanercept (IL10, TNFA ), infliximab (TNFRSF1B, TNFA, FCGR2A/3A), and other drugs [83]. In other words, today, it is possible to choose about 10–15 genes, the polymorphisms of which can serve as a prognostic criterion for Caucasians in RA therapy.

## 6. Conclusions

Recently, the results of a GWAS—a study aimed to determine personalised RA therapy based on the genetic polymorphisms associated with sensitivity to FDA-approved antirheumatic drugs—have been published. Moreover, epigenetic changes (including microRNA polymorphism and expression) altering the disease complication risk and response to treatment should be taken to account, along with the germline variants assessment for RA predisposition and therapy response [96,97]. In that way, researchers are striving to develop a genetic testing algorithm for DMARD therapy personalisation to make RA treatment more clinically- and cost-effective. Thus, the published results of original scientific studies, reviews, and meta-analyses allow us to characterise the genetic component of RA predisposition in various populations and emphasise specific variants to be analysed for the most effective treatment regimens.

## Figures and Tables

**Table 1:** Polymorphisms (non-HLA genes) associated with rheumatoid arthritis (RA) in mixed populations according to meta-analyses data.

Protein Product	Gene	Polymorphism	Allele/Genotype Associated with RA	Reference
Protein tyrosine phosphatase, nonreceptor type 22	*PTPN22*	rs2476601 rs2488457	T C	[30,31,32,36]
Peptidyl arginine deiminase 4	*PADI4*	rs11203367 rs884871 rs2240340	T G A	[37]
Tumor necrosis factor, alpha-induced protein 3 (A20)	*TNFAIP3*	rs2230926 rs5029937	G T	[28]
Cytotoxic T-lymphocyte associated protein 4	*CTLA4*	rs231775	GG	[38]
Signal transducer and activator of transcription 4	*STAT4*	rs7574865	T	[29]
C-C motif chemokine receptor 6	*CCR6*	rs3093024	A	[24]
C-C motif chemokine ligand 2 (monocytes chemo-attractant)	*CCL2*	rs1024611	G	[39]
Autoimmune regulator (transcription factor)	*AIRE*	rs2075876 rs760426	A G	[35]
Methylene tetrahydrofolate reductase	*MTHFR*	rs1801133 rs1801131	T CC	[40,41]
Interleukin 10	*IL10*	rs1800896 rs3021097 rs1800872	AA TT AA	[42]
Interleukin 23 receptor	*IL23R*	rs11209026 rs10489629 rs1343151	AA G A	[23,43]
Interleukin 17	*IL17*	rs2275913	AA	[44]
Tumour necrosis factor receptor type 2	*TNFR2*	rs1061622	GG	[45]
Transforming growth factor beta and its receptors	*TGFB*	rs1800470 rs1800469	TT TT	[46]

**Table 2:** Polymorphisms (non-HLA genes) associated with disease-modifying antirheumatic drugs (DMARD) efficacy, according to meta-analyses and multicenter studies data.

Drug Class	Gene	Polymorphism	Association	Reference
Cytostatic agents	*MTHFR*	rs1801133	T-allele–MTX toxicity	[75,76]
	*ATIC*	rs7563206 rs2372536	T-allele G allele–MTX nonresponders	[77,78]
	*TYMS*	rs2244500 rs2847153 rs3786362	AA genotype A-allele G-allele–MTX efficacy	[78]
	*RFC1*	rs1051266	AA genotype–MTX efficacy	[70]
	*ABCB1*	rs1045642	C-allele–MTX efficacy	[79]
	*DHODH*	rs3213422	AA genotype–leflunomide efficacy	[67]
IL6 inhibitors	*IL6R*	rs12083537 rs11265618	AA genotype CC genotype–tocilizumab better response	[80,81]
Glucocorticoids	*GLCCI1* *GR*	rs37972 rs41423247 rs6195	T-allele–decreased sensitivity G-allele–increased sensitivity to transition therapy	[82]
TNF inhibitors	*TNFRSF1B*	rs1061622	G-allele–increased sensitivity	[83]
	*TNFA*	rs1800629 rs361525	A-alleles–anti-TNF agent efficacy	[11]
	*FCGR2A*	rs1801274	HH + HR genotype–adalimumab efficacy	[84]
	*PTPRC*	rs10919563	A-allele–decreased sensitivity	[84]
	*TLR1* *TLR5*	rs4833095 rs5744174	CC genotype; C-allele–anti-TNF agent efficacy	[85]
	*NUBPL*	*rs2378945*	Minor allele–decreased etanercept sensitivity	[86]

MTX—methotrexate, IL6 – interleukine 6, TNF—tumor necrosis factor.
